# Using a Hybrid Model to Forecast the Prevalence of Schistosomiasis in Humans

**DOI:** 10.3390/ijerph13040355

**Published:** 2016-03-23

**Authors:** Lingling Zhou, Jing Xia, Lijing Yu, Ying Wang, Yun Shi, Shunxiang Cai, Shaofa Nie

**Affiliations:** 1 Department of Epidemiology and Biostatistics, School of Public Health, Tongji Medical College, Huazhong University of Science and Technology, Wuhan 430001, China; zllgwy@126.com (L.Z.); yulijing321@outlook.com (L.Y.); wangying@whu.edu.cn (Y.W.); shiyuntj@hust.edu.cn (Y.S.); 2 Department of Information, Research Institute of Field Surgery, Daping Hospital of Third Military Medical University, Chongqing 400042, China; 3 Institute of Parasitic Disease Control, Hubei Provincial Center for Disease Control and Prevention, Wuhan 430079, China; xiaj0608@163.com

**Keywords:** schistosomiasis, forecasting, ARIMA model, NARNN model, hybrid model

## Abstract

*Background*: We previously proposed a hybrid model combining both the autoregressive integrated moving average (ARIMA) and the nonlinear autoregressive neural network (NARNN) models in forecasting schistosomiasis. Our purpose in the current study was to forecast the annual prevalence of human schistosomiasis in Yangxin County, using our ARIMA-NARNN model, thereby further certifying the reliability of our hybrid model. *Methods*: We used the ARIMA, NARNN and ARIMA-NARNN models to fit and forecast the annual prevalence of schistosomiasis. The modeling time range included was the annual prevalence from 1956 to 2008 while the testing time range included was from 2009 to 2012. The mean square error (MSE), mean absolute error (MAE) and mean absolute percentage error (MAPE) were used to measure the model performance. We reconstructed the hybrid model to forecast the annual prevalence from 2013 to 2016. *Results*: The modeling and testing errors generated by the ARIMA-NARNN model were lower than those obtained from either the single ARIMA or NARNN models. The predicted annual prevalence from 2013 to 2016 demonstrated an initial decreasing trend, followed by an increase. *Conclusions*: The ARIMA-NARNN model can be well applied to analyze surveillance data for early warning systems for the control and elimination of schistosomiasis.

## 1. Introduction

Schistosomiasis is an acute and chronic, neglected tropical parasitic disease that is globally distributed in 78 countries, including Africa, Asia, the Middle East, and South America [1]. The World Health Organization (WHO) has reported that at least 249 million people required treatment in 2013, while the actual number of treated people in that year was only 42.1 million. This great deficit underscores both the profound impact that schistosomiasis has on worldwide populations and the fact that it is often neglected.

In China, HIV/AIDS, tuberculosis, hepatitis B and schistosomiasis have been listed the top 4 infectious diseases [2]. Although prioritized for control and elimination since 2004, schistosomiasis is still considered a major public health problem today. The government has undertaken some highly effective and comprehensive strategies to manage schistosomiasis, which include developing and implementing the “Outline of mid- and long-term national programme on control and prevention of schistosomiasis (2004–2015)” as well as the “Outline of key project planning for comprehensive management of schistosomiasis (2009–2015)” [3]. A total of 184,943 cases of schistosomiasis were estimated and nine acute cases were reported in 2013 [4]. As compared to data from 2009 [5], the reduction rates of total cases were 49.44% and 88.31%, respectively, indicating that the national endemic situation has reached its lowest level in Chinese history. Despite these promising trends, it will still take a great deal of time to achieve the goal of complete blockage of schistosomiasis transmission and its resulting total elimination as written in the document entitled “Criteria for control and elimination of schistosomiasis” [6]. The length of time required for its eradication is due in large part to the fact that the key natural and social risk factors for transmission have not fundamentally changed. 

To better aid the ultimate goal of elimination, forecasting techniques can be used to analyze the occurrences, development, and future trends of schistosomiasis, so as to guide the basic measures for control and prevention of schistosomiasis. Forecasting is a particularly powerful tool to facilitate the development of effective control strategies for schistosomiasis that has frequent fluctuations. More recently, hybrid forecasting models have been extensively applied in the field of communicable disease with high predictive performance [7,8,9,10]. Autoregressive integrated moving average (ARIMA) model is a classical linear time series forecasting model, which has been widely utilized to predict a time series, including many communicable diseases such as tuberculosis [11], malaria [12], hepatitis [13], hemorrhagic fever [14], dengue fever [15] and influenza [16]. For nonlinear time series forecasting problems, artificial neural networks (ANNs) have been widely applied in many different fields [17,18,19,20,21], due to their high fault tolerance performance, self-learning, self-organization, and ability to approximate any sort of arbitrary nonlinear function [22,23]. Although both ARIMA and ANNs are superior in modeling a variety of problems, neither can be used indiscriminately in every type of forecasting situation. Theoretically, combining both the ARIMA and ANNs models would allow for an improved method for forecasting.

Our team has proposed a new hybrid approach combining both the ARIMA and the nonlinear autoregressive neural network (NARNN) models to successfully forecast the prevalence of schistosomiasis in humans of Qianjiang City, China [24]. The purpose of this paper is to further validate the feasibility of the ARIMA-NARNN hybrid model in prediction of schistosomiasis infections.

## 2. Methodology

### 2.1. Data Sources

Schistosomiasis can be found around Hubei Province, China. In 2013, the prevalence of schistosomiasis in humans of Hubei Province was under 1.00%, reaching the national criteria of transmission control. Nonetheless, there are still two major challenges. First, areas infested with *Oncomelania* snails were about 76,600–77,400 hm^2^ in 2012, ranking it first in all schistosomiasis endemic provinces of China. Second, cattle are not customarily raised in captivity in the most endemic areas of Hubei Province, leading to rampant sanitation and hygiene problems due to animal waste. These two conditions make adequate control of schistosomiasis infections increasingly difficult. 

Yangxin County is located in the southeast region of Hubei Province and has a climate characterized by subtropical monsoon seasons. The environment and climate are very suitable for the breeding of snails, which has led to endemic levels of schistosomiasis in Yangxin County. It has historically been one of the hardest schistosomiasis hit in Hubei Province. Taking into account the availability and completeness of data collection, we chose Yangxin County as our study area.

According to Chinese criteria, schistosomiasis is a statutory B class infectious disease and has been monitored according to the “national surveillance scheme of schistosomiasis” [25]. One national surveillance point and nine provincial surveillance points had been set up in Yangxin County, similar to those in the city of Qianjiang City [24]. Human prevalence of schistosomiasis is one of the surveillance indicators. We obtained the annual report data of prevalence from 1956 to 2012 from the Schistosomiasis Prevention and Control Office of Yangxin County (Table S1). 

### 2.2. The ARIMA Model

The ARIMA (*p*, *d*, *q*) model [26] is made up of three parts, where *p* is the order of auto-regression (AR), *d* is the order of regular differencing, and *q* is the order of moving average (MA). Stationarity is a necessary condition in building an ARIMA model and differencing is often used to stabilize the time series data. Lagged scatter-plots, autocorrelation function (ACF), partial autocorrelation function (PACF) plots, or augmented dickey-fuller unit root (ADF) test are used to identify whether or not the time series is stationary. The modeling process we used included three iterative steps of model identification, parameter estimation, and diagnostic checking. This three-step model building process was typically repeated several times until a satisfactory model was finally selected. 

We used SAS Software Version 9.2 (North Carolina State University, Raleigh, NC, USA) to develop the ARIMA model. The SAS procedure can automatically provide the minimal value of Bayesian information criterion (BIC) and estimate the parameters by the conditional least square method to choose the optimal model. The *Ljung-Box Q-test* for diagnostic checking helped to identify whether the residual series was the white noise. The white noise series would indicate that the information has been sufficiently extracted, allowing the model to conduct the predictive analysis. In this study, for the prediction performance comparison, the modeling set used was from 1956 to 2008 and the testing set from 2009 to 2012. We constructed the ARIMA model using the entire 57 years data set in order to forecast the future prevalence of schistosomiasis from 2013 to 2016. This information was then used to compute the residual series as the target series of NARNN. 

### 2.3. The NARNN Model

ANNs are computing systems containing many simple nonlinear units interconnected by links [23]. NARNN, which is one of the dynamic ANNs based on the feed-forward, back-propagation network (FFBP) with feedback layers [27], can predict a simple time series given past values of the same time series. In this paper, we utilized the Neural Network Toolbox of MATLAB Version 7.11 (R2010b, MathWorks, Natick, MA, USA). In this tool, NARNN incorporates a default two-layer FFBP with a sigmoid transfer function in the hidden layer, a linear transfer function in the output layer, and utilization of the *Levenberg-Marquardt* algorithm in training. 

Using this tool, dynamic NARNN processing was determined on the best form and the command-line script was generated automatically. Before modeling, we divided the target series into a training subset, validation subset, and testing subset using the default random division function. The ratios for training, validation, and testing were set to 0.80, 0.10 and 0.10, respectively. The training subset was used for computing the gradient and updating the network weights and biases. The error on the validation subset was monitored during the training process. When the network began to overfit the data, the error on the validation subset typically rose. The network weights and biases were saved at the minimum of the validation set error. When the validation error increased for a specified number of iterations (we used six iterations in our training), the training was stopped, and the weights and biases at the minimum of the validation error were returned. We were able to choose the relative optimal model by using the error autocorrelation plot, the time series response plot, the mean square error (MSE), and the correlation coefficient (R) between outputs and targets as the indices. We used trial and error to adjust the parameters feedback delays and hidden units until an optimal model was generated. In the study, depending on our experience, the hidden units and feedback delays range included from 10–18 and 3–7 respectively. In total of 45 architectures were tested one by one.

### 2.4. Developing the Hybrid ARIMA-NARNN Model

We discovered linear relationships in the first stage of modeling the original prevalence data with the ARIMA model. The estimation of original prevalence yields the forecast value *L_t_*. The ARIMA model was then used to generate the residuals *e_t_*. In the second stage, the NARNN model was used to model the nonlinear relationships existing in the residuals. The estimation of *e_t_* yields the forecast value ${\overset{\wedge }{N}}_{t}$.

The combined forecasting values of the time series were as follows: ${\overset{\wedge }{y}}_{t}=\overset{\wedge }{L_{t}}+{\overset{\wedge }{N}}_{t}$, where ${\overset{\wedge }{y}}_{t}$ was the predicted value by the ARIMA-NARNN model at time t, $\overset{\wedge }{L_{t}}$ denoted the estimation of linear component from the ARIMA model, and ${\overset{\wedge }{N}}_{t}$ denoted the residuals predicted by the NARNN model. In the study, 4-step-ahead prediction was performed to track the annual prevalence of schistosomiasis from 2013 to 2016.

### 2.5. Performance Statistics Index

In order to compare the forecasting performance of the ARIMA, NARNN and ARIMA-NARNN models, three indices were used to evaluate prediction accuracy: the MSE, mean absolute error (MAE), and mean absolute percentage error (MAPE). Their calculation formulas were as follows:

(1) $$MSE=\frac{1}{n}\sum_{t=1}^{n}{\left(y_{t}-\overset{\wedge }{y_{t}}\right)}^{2}$$

(2) $$MAE=\frac{1}{n}\sum_{t=1}^{n}\left|y_{t}-\overset{\wedge }{y_{t}}\right|$$

(3) $$MAPE=\frac{1}{n}\sum_{t=1}^{n}\frac{\left|y_{t}-\overset{\wedge }{y_{t}}\right|}{y_{t}}$$

where $y_{t}$ and $\overset{\wedge }{y_{t}}$ denote the original and the predicted prevalence at time t respectively, and n is the number of predictions. Good fitness and prediction performance is demonstrated with these three indices showing as small a value as possible.

## 3. Results

### 3.1. ARIMA Model Analysis

**Figure 1:**
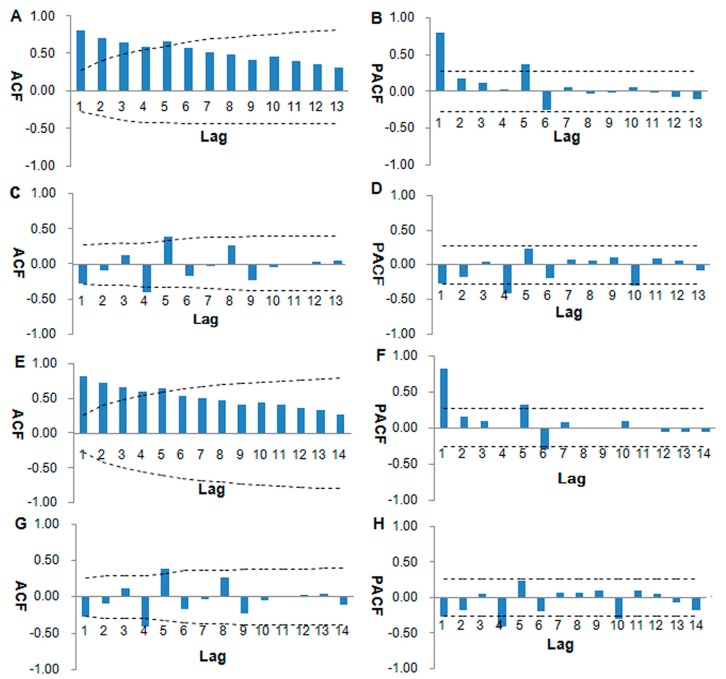
Autocorrelation function (ACF) and partial autocorrelation function (PACF) plots of original prevalence series (OS). (**A**) and (**B**) show ACF and PACF plots of OS (1956–2008). (**C**) and (**D**) show ACF and PACF plots after one order of differencing (1956–2008). (**E**) and (**F**) show ACF and PACF plots of OS (1956–2012). (**G**) and (**H**) show ACF and PACF plots after one order of differencing (1956–2012). Dotted lines indicate 95% confidence intervals.

The ACF and PACF plots of different original prevalence series (OS) are displayed in Figure 1, the Figure 1A,B,E, and F which collectively suggest that the series was non-stationary. As shown in Figure 1C,D,G, and H, most of the correlations fell around zero within their 95% confidence intervals after one order of differencing, suggesting that the series achieved stationarity. 

The results of the ADF test are showed in Table 1. As all the *p*-values were less than 0.05, we concluded that there was no unit root, which provided further confirmation that the differenced series was stationary.

We found the minimum BIC (5, 7) = 0.7895 (using 1956–2008 as the modeling set) and BIC (5, 7) = 0.6716 (using 1956–2012 as the modeling set), resulting in an order of auto-regression of *p* = 5 and the order of moving average of *q* = 7. The results of the parameter estimations are shown in Table 2. 

All of the estimated parameter values are statistically significant (*p* < 0.05).The autocorrelation checks of residuals are presented in Table 3. All the *p*-values > 0.05, showing that the residuals were all a white noise series. Based on these residual results, we concluded that the model could be used to forecast future prevalence of schistosomiasis. 

The predicted prevalence of the testing set, spanning from 2009 to 2012, was 0.40%, 0.99%, 0.62% and −0.96% respectively. The values of MSE, MAE and MAPE are presented in Table 4. The predicted future prevalence obtained from the ARIMA ((4), 1, (5)) model, for years 2013 to 2016 was −1.03%, −0.80%, −1.27% and −0.54%, respectively. We then computed the residual series (RS) from 1956 to 2008 and the new residual series (NRS) from 1956 to 2012, which were subsequently used as the target series of the NARNN model.

### 3.2. NARNN Model Analysis

The parameters for the optimum NARNN model are shown in Table 5, target series OS with hidden units 16 and delays 5, RS with hidden units 14 and delays 6, and NRS with hidden units 14 and delays 5. All R values were greater than 0.85. All MSE values of the training, validation, and testing subsets were found to be relatively small. 

**Figure 2:**
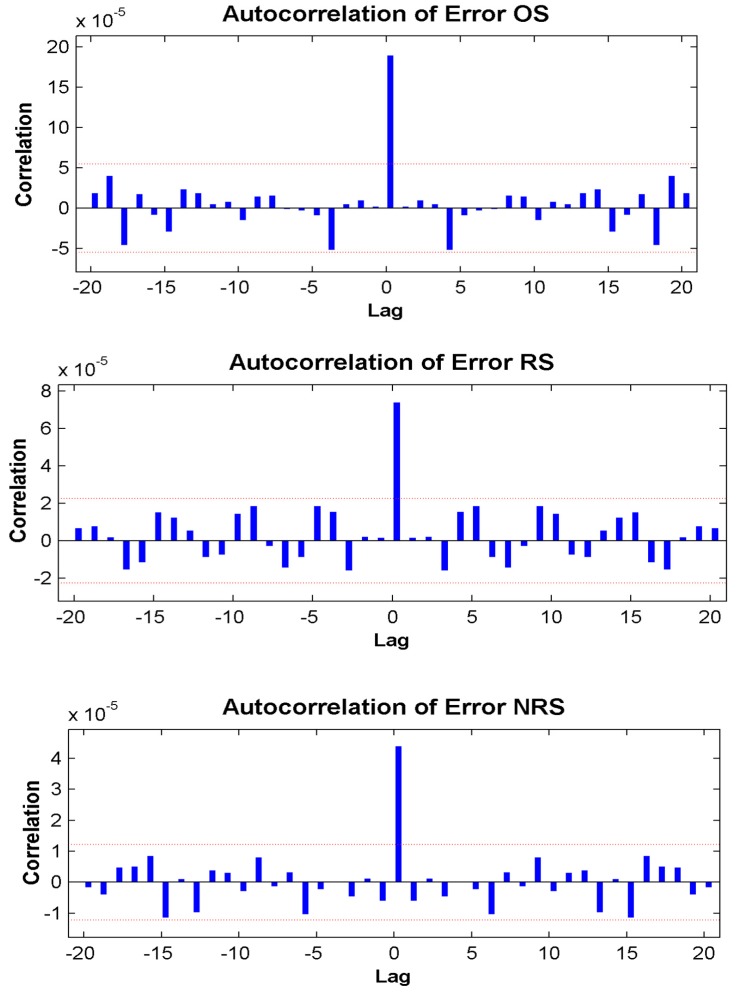
Error autocorrelation plots of different target series from appropriate NARNN model. The red dotted line indicate 95% confidence intervals. All the coefficients fell within the 95% confidence limits with the exception of the autocorrelation coefficient at zero lag, indicating that the model reliably corresponds to the data. OS = original prevalence series, RS = residual series, NRS = new residual series.

The error autocorrelation function plots are displayed in Figure 2. The error autocorrelation was one of the evaluation parameters in the modeling process. As shown in Figure 2, the correlations except for the one at zero lag, all fell within the 95% confidence limits, demonstrating that the model was adequate. 

**Figure 3:**
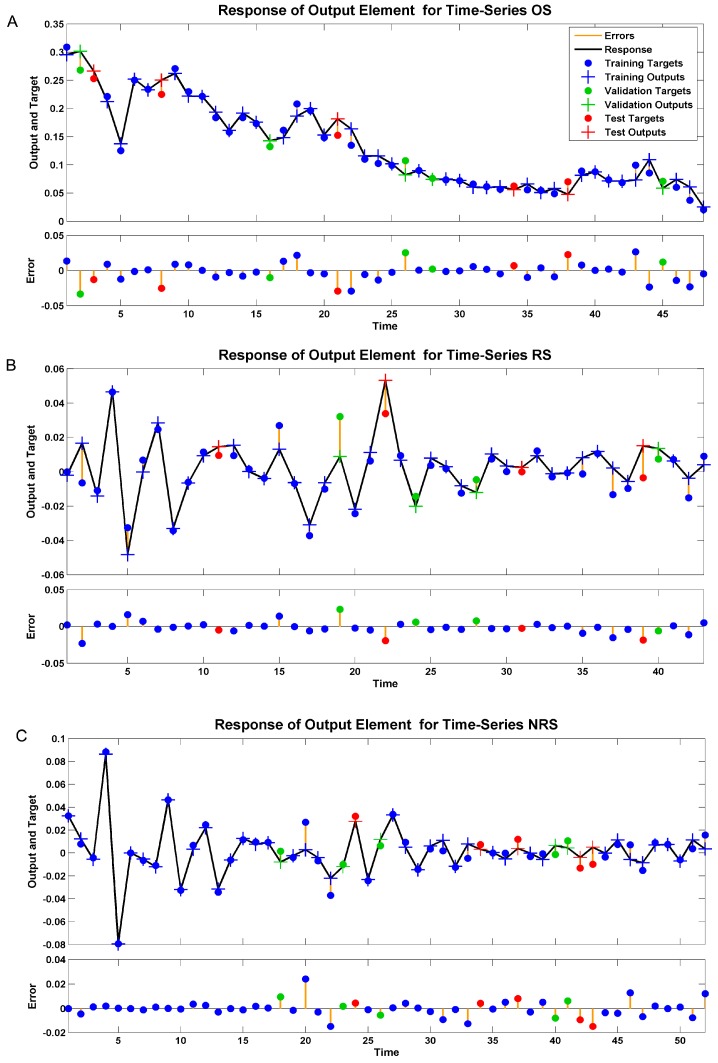
Time series response plots of different target series from the appropriate NARNN model. (**A**–**C**) display the inputs, targets, and errors versus time and also give which time points were selected for training, testing, and validation.

The time series response plots are displayed in Figure 3. The outputs are distributed evenly on both sides of the response curve and the errors are small in the training, testing, and validation subsets. These provided further confirmation that we had chosen the appropriate model.

### 3.3. ARIMA-NARNN Model Analysis

**Figure 4:**
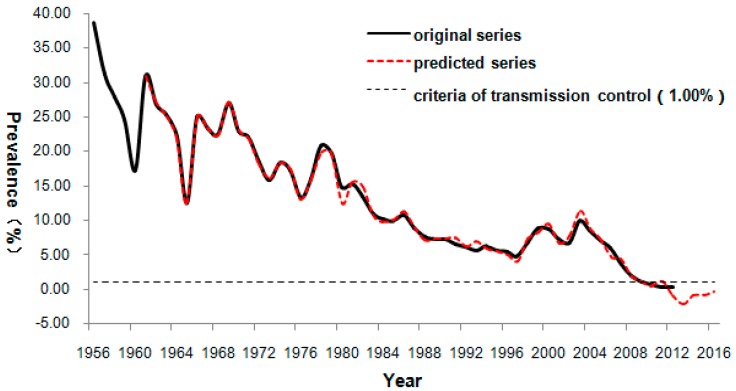
The change trend plot of the prevalence of schistosomiasis in humans of Yangxin County. The black line represents the original prevalence series (1956–2012) and the red line represents the predicted prevalence series (1961–2016) from the ARIMA-NARNN model. The black dotted line gives the criteria of schistosomiasis transmission control in humans.

The predicted values by the ARIMA-NARNN model from 2009 to 2012 were 1.54%, 0.09%, 0.34% and 0.38% respectively. The values of future forecasting from 2013 through 2016 were −2.11%, −0.90%, −0.84%, and −0.37%, respectively. The predicted change trend from the hybrid model is shown in Figure 4. The curves of the original and predicted series are very similar, indicating that the hybrid model was well fitted to the data of schistosomiasis prevalence in humans of Yangxin County.

### 3.4. Comparison of Results from Forecasting Performance

Table 4 presents the differences in modeling error (from 1956 to 2008) and testing error (from 2009 to 2012) between the original and predicted values using the single ARIMA, single NARNN, and combined ARIMA-NARNN models. As shown, the hybrid model was the best model, with the lowest MSE, MAE, and MAPE. 

## 4. Discussion

In this study, we sought to construct a single ARIMA model, a single NARNN model, and a combined ARIMA-NARNN hybrid model based on the data of human prevalence of schistosomiasis in Yangxin County. The modeling MSE, MAE, and MAPE were reduced by 52.03%, 44.56% and 60.93% and the corresponding testing error fell by 80.26%, 59.09% and 71.63% respectively as compared to using the ARIMA model alone. When compared to the single NARNN model, the modeling MSE, MAE, and MAPE were decreased by 73.89%, 37.89% and 35.80% and the corresponding testing error reduced by 67.58%, 55.00% and 65.67%, respectively. Similar to the previous forecasting study in Qianjiang City, the hybrid model achieved the lowest MSE, MAE and MAPE among the three models, again demonstrating that the combined ARIMA-NARNN model provided a reliable hybrid forecasting approach to predict the prevalence of schistosomiasis in humans. Comparison of prediction accuracy of ARIMA-NARNN model in the two areas, in Yangxin County showed that the modeling error was significantly higher than that of Qianjiang City, but the testing error was lower than that of Qianjiang City [24]. The different characteristics of the data from different areas lead to different prediction accuracies of the hybrid model, therefore it is unreasonable to judge if the hybrid model is more suitable for certain area only by comparing the errors. In addition, in terms of the prediction accuracies of the single ARIMA and single NARNN models, they were different between the two areas. In Yangxin County, the forecasting performance of NARNN model was better than ARIMA model, however, in Qianjiang City, the forecasting performance of ARIMA model is slightly better than NARNN model.

As shown in Figure 4, the prevalence of schistosomiasis often fluctuated during the 57 years from 1956 to 2012, but with a general decrease in overall development. The 10-year implementation of the World Bank Loan Project for Schistosomiasis Control (1992–2001) that provided sufficient funds resulted in great achievements [28]. Although the prevalence decreased to a lower level in 1997 (4.89%), there was a subsequent increase afterwards, reaching a peak value of 9.97% in 2003. The uptick is likely due to multiple causes, including the end of the loan project, the flooding of the Yangtze River Watershed, increased population mobility, global warming, as well as ecosystem changes caused by the construction of the Three Gorges Dams and the South–North Water Conversion Project [29,30,31]. Thereafter, Chinese government took action with a series of integrated control strategies and the prevalence decreased yearly since 2004 [32]. As a result of the implementation of this new strategy, by the end of 2010, the prevalence of schistosomiasis was reduced to 0.65%. This met the criteria for transmission control of human schistosomiasis in China [6], with a prevalence of less than 1.00%. The predicted prevalence from 2013 to 2016 was also under this criterion. However, these predicted values showed a slight increase which could be an alert for policymakers to strengthen our current control programs in order to prevent a rebound of the disease and further achieve the goal of schistosomiasis elimination. Although the baseline data and the predicted prevalence of schistosomiasis from 2013 to 2016 in Qianjiang City and Yangxin County suggest that the developments of the epidemic trends of schistosomiasis are different between the two areas, the ARIMA-NARNN hybrid model is still applicable in the both places.

In our proposed hybrid model, the linear ARIMA model and the nonlinear NARNN model were jointly used, aimed at capturing different forms of the relationship in the time series data so as to improve forecasting performance. The empirical results clearly suggest that the ARIMA-NARNN hybrid model is able to outperform each component model used in isolation. In the first modeling stage, the ARIMA model dealt with the non-stationary linear component of the original prevalence series. The best ARIMA models we have established were ARIMA ((4), 1, (5)), which showed that the predicted values at year t depended not on the previous year t-4, but on the random error at year t-5. Based on the modeling data 1956–2008 and 1956–2012, the constructed ARIMA models had the same order parameters and the estimated model parameters were also very close. There was similar result to the study of Qianjiang. This could be due to the steady downward trend without fluctuation from 2009 to 2012 in both of these two areas, which had little influence on the ARIMA model. Although developing the ARIMA model requires constantly rejoining the new actual values, when the characteristics of the modeling data are similar to those in this study, we can also try to build the ARIMA model, ignoring the absence of the last few actual time values. In the second stage, the NARNN model dealt with nonlinearity by modeling the residual series from the ARIMA model. Target series OS with delays 5, RS with delays 6, and NRS with delays 5 indicates that the predicted residuals of the corresponding OS, RS, and NRS at year t depended on the previous 5, 6 and 5 years’ values, respectively. We then constructed the ARIMA-NARNN model with the finally predicted yearly prevalence from 2013 to 2016 at less than zero. To be clear, in the forecasting analysis, the negative values represent the predicted trend rather than an actual value. If the modeling data were extremely small, the subsequent corresponding predictions would be negative. 

In recent years, hybrid models combining linear and nonlinear components for time series forecasting have been extensively applied [33,34,35,36,37]. Given the results of our modeling analysis, we want to highlight the following advantages presented by this work. First, the SAS procedure can automatically generate the minimum BIC, which breaks up the order process to help set up the ARIMA model. Second, NARNN model is generally more powerful than static networks developed in some previous studies of time series forecasting, such as the multilayer perceptron (MLP), back-propagation neural network (BP), radial basis function neural network (RBF), probabilistic neural network (PNN) and generalized regression neural network (GRNN) [20,38,39,40]. The NARNN model which can be trained to learn time-varying patterns is applicable to the data set in this study. We performed necessary calculations with an easy-to-use graphical environment in the neural network tool of MATLAB, which ultimately allowed us to easily design the NARNN model. Our proposed hybrid model is easier to be mastered than the other models found in the references [33,34,35,36,37], and can improve the applicablity to the grass-roots workers in control of schistosomiasis. In addition, time series forecasting is a far easier approach than models associated with the risk factors of schistosomiasis infection including the distribution of *Oncomelania*, socio-economic factors, ecology environment, life style, diagnostic tools and therapeutic methods [41,42]. However, by eliminating these factors, the time series forecasting analysis we currently have is incomplete. Another constraint of time series forecasting is its limited ability to extrapolate—the longer the forecasting time, the lower the prediction accuracy. 

## 5. Conclusions

Our study showed that the combined ARIMA-NARNN model is a reliable tool to forecast the prevalence of schistosomiasis in humans. Further studies will be needed to develop synthetic approaches combining various factors and methods of different types to improve the ability of early warning and prediction of schistosomiasis. Importantly, this model is by no means isolated to use in schistosomiasis prediction, and could easily be adapted to forecast other communicable diseases.

## Figures and Tables

**Table 1:** Augmented dickey-fuller unit root (ADF) test of different modeling sets.

Type	Lag	1956–2008		1956–2012	
		*t*	*p* ^a^	*t*	*p* ^a^
Zero Mean	0	−9.39	<0.0001	−9.73	<0.0001
	1	−6.74	<0.0001	−6.98	<0.0001
Single Mean	0	−9.60	0.0001	−9.98	0.0001
	1	−7.04	0.0001	−7.32	0.0001
Trend	0	−9.54	<0.0001	−9.92	<0.0001
	1	−6.98	<0.0001	−7.27	<0.0001

Note: ^a^, It was considered that there was nonexistent unit root (*p* < 0.05).

**Table 2:** Parameter estimations of different modeling sets from ARIMA model.

Modeling Set	Parameter	Estimate	Standard Error	*t*	*p* ^a^	Lag
1956–2008	AR1,1	−0.33659	0.13474	−2.50	0.0158	4
	MA1,1	−0.59063	0.11667	−5.06	<0.0001	5
1956–2012	AR1,1	−0.33529	0.12856	−2.61	0.0118	4
	MA1,1	−0.58920	0.11144	−5.29	<0.0001	5

Note: ^a^, Parameter estimations were considered statistically significant (*p* < 0.05).

**Table 3:** The white noise check of residuals from different modeling sets.

Lag	1956–2008		1956–2012	
	*χ*^2^	*p* ^a^	*χ*^2^	*p* ^a^
6	7.19	0.1262	7.66	0.1050
12	13.27	0.2090	13.96	0.1747
18	15.04	0.5219	15.77	0.4691
24	16.92	0.7679	17.66	0.7261

Note: ^a^, The residual series was a white noise series (*p* > 0.05).

**Table 4:** Prediction results of three models.

Year	Original Values (%)	Pridicted Values (%)		
		ARIMA	NARNN	ARIMA-NARNN
2009	1.13	0.40	1.75	1.55
2010	0.65	1.00	1.44	0.09
2011	0.42	0.62	1.01	0.34
2012	0.39	−0.96	0.80	0.38
Error				
Modeling	MSE(×10 ^-4^)	2.8272	2.1089	0.7381
	MAE	0.0123	0.0095	0.0059
	MAPE	0.1223	0.1056	0.0678
Testing	MSE(×10 ^-4^)	0.6267	0.3816	0.1237
	MAE	0.0066	0.0060	0.0027
	MAPE	1.2791	1.0570	0.3629

**Table 5:** Optimum network parameters of different target series.

Target Series ^a^	Hidden Units	Delays	MSE ^b^ (×10^−4^)			R ^c^
			Training	Validation	Testing	
OS	16	5	1.2671	4.0469	4.4604	0.9838
RS	14	6	0.5022	1.6596	1.8805	0.8828
NRS	14	5	0.3911	0.4463	0.8199	0.9579

Notes: ^a^, OS = original prevalence series, RS = residual series, NRS = new residual series; ^b^, MSE = mean square error; ^c^, R = correlation coefficient.

## Supplementary Materials

The following are available online at www.mdpi.com/1660-4601/13/4/355/s1, Table S1: Annual prevalence of schistosomiasis in Yangxin County (1956–2012).
