# Supplementary material for: Using a Hybrid Model to Forecast the Prevalence of Schistosomiasis in Humans
Source: Int J Environ Res Public Health. 2016 Mar 23;13(4):355. doi: 10.3390/ijerph13040355 (PMC4847017; doi:10.3390/ijerph13040355)
Supplement: Supplementary file 1 [file ijerph-13-00355-s001.pdf]

Supplementary Materials: Using a Hybrid Model to Forecast the Prevalence of Schistosomiasis in Humans

Lingling Zhou, Jing Xia, Lijing Yu, Ying Wang, Yun Shi, Shunxiang Cai and Shaofa Nie

Table S1. Annual prevalence of schistosomiasis in Yangxin County (1956–2012).

Year	Prevalence of Schistosomiasis (%)	Year	Prevalence of Schistosomiasis (%)
1956	38.65	1985	9.94
1957	31.27	1986	10.76
1958	27.98	1987	9.02
1959	24.56	1988	7.61
1960	17.3	1989	7.37
1961	30.9	1990	7.2
1962	26.8	1991	6.58
1963	25.31	1992	6.15
1964	22.13	1993	5.68
1965	12.54	1994	6.27
1966	25.06	1995	5.58
1967	23.41	1996	5.47
1968	22.51	1997	4.89
1969	27.1	1998	7.01
1970	23.01	1999	8.9
1971	22.17	2000	8.77
1972	18.41	2001	7.36
1973	15.83	2002	6.86
1974	18.4	2003	9.97
1975	17.34	2004	8.57
1976	13.27	2005	7.07
1977	16.14	2006	6.02
1978	20.82	2007	3.72
1979	19.63	2008	2.05
1980	14.82	2009	1.13
1981	15.24	2010	0.65
1982	13.46	2011	0.42
1983	11.01	2012	0.39
1984	10.25		

© 2016 by the authors; licensee MDPI, Basel, Switzerland. This article is an open access article distributed under the terms and conditions of the Creative Commons by Attribution (CC-BY) license (http://creativecommons.org/licenses/by/4.0/).
